# DArT markers: diversity analyses, genomes comparison, mapping and integration with SSR markers in *Triticum monococcum*

**DOI:** 10.1186/1471-2164-10-458

**Published:** 2009-09-30

**Authors:** Hai-Chun Jing, Carlos Bayon, Kostya Kanyuka, Simon Berry, Peter Wenzl, Eric Huttner, Andrzej Kilian, Kim E Hammond-Kosack

**Affiliations:** 1 Centre for Sustainable Pest and Disease Management, Department of Plant Pathology and Microbiology, Rothamsted Research, Harpenden, Hertfordshire, AL5 2JQ, UK; 2 Nickerson-Advanta, Woolpit Business Park, Woolpit, Bury St Edmunds, Suffolk, IP30 0RA, UK; 3 Diversity Arrays Technology P/L and Triticarte Pty Ltd, 1 Wilf Crane Cr., Yarralumla, Canberra, ACT 2600, Australia; 4 Centre for Bioenergy Plants Research and Development, Institute of Botany, Chinese Academy of Sciences, Beijing, 100093, PR China

## Abstract

**Background:**

*Triticum monococcum *(2*n *= 2*x *= 14) is an ancient diploid wheat with many useful traits and is used as a model for wheat gene discovery. DArT (Diversity Arrays Technology) employs a hybridisation-based approach to type thousands of genomic loci in parallel. DArT markers were developed for *T. monococcum *to assess genetic diversity, compare relationships with hexaploid genomes, and construct a genetic linkage map integrating DArT and microsatellite markers.

**Results:**

A DArT array, consisting of 2304 hexaploid wheat, 1536 tetraploid wheat, 1536 *T. monococcum *as well as 1536 *T. boeoticum *representative genomic clones, was used to fingerprint 16 *T. monococcum *accessions of diverse geographical origins. In total, 846 polymorphic DArT markers were identified, of which 317 were of *T. monococcum *origin, 246 of hexaploid, 157 of tetraploid, and 126 of *T. boeoticum *genomes. The fingerprinting data indicated that the geographic origin of *T. monococcum *accessions was partially correlated with their genetic variation. DArT markers could also well distinguish the genetic differences amongst a panel of 23 hexaploid wheat and nine *T. monococcum *genomes. For the first time, 274 DArT markers were integrated with 82 simple sequence repeat (SSR) and two morphological trait loci in a genetic map spanning 1062.72 cM in *T. monococcum*. Six chromosomes were represented by single linkage groups, and chromosome 4A^m ^was formed by three linkage groups. The DArT and SSR genetic loci tended to form independent clusters along the chromosomes. Segregation distortion was observed for one third of the DArT loci. The *Ba *(black awn) locus was refined to a 23.2 cM region between the DArT marker locus *wPt-2584 *and the microsatellite locus *Xgwmd33 *on 1A^m^; and the *Hl *(hairy leaf) locus to a 4.0 cM region between DArT loci *376589 *and *469591 *on 5A^m^.

**Conclusion:**

DArT is a rapid and efficient approach to develop many new molecular markers for genetic studies in *T. monococcum*. The constructed genetic linkage map will facilitate localisation and map-based cloning of genes of interest, comparative mapping as well as genome organisation and evolution studies between this ancient diploid species and other crops.

## Background

*Triticum monococcum *(2*n *= 2*x *= 14), generally known as einkorn wheat, is an ancient diploid species domesticated in the Fertile Crescent ~10,000 years ago ([1]. This species dominated human farming activities in the Neolithic period. However, the cultivation area gradually decreased during the Bronze Age due to domestication of tetraploid and hexaploid wheat [2]. In modern times *T. monococcum *remains cultivated at low scale only in the mountainous areas of several Mediterranean countries. This species has not been bred intensively and therefore retained its genetic diversity [3].

Wild relatives of hexaploid wheat are known to be important sources of traits for wheat genetic improvement. *T. monococcum *(A^m^A^m^) is closely related to *T. urartu *(A^u^A^u^), the donor of the A-genome of hexaploid wheat [4-6]. Recently, *T. monococcum *has gradually been recognised as an attractive diploid model for exploitation of useful traits, discovery of novel genes and variant alleles, and functional genomics. Many traits have been examined in *T. monococcum *which can be useful for modern wheat breeding [7-9]. *T. monococcum *has also been successfully used for gene discovery in a subgenome map-based cloning approach, as exemplified by the cloning of the leaf rust resistance gene *Lr10 *[10,11], the vernalisation genes *VRN1 *and *VRN2 *[12,13], the domestication locus *Q *[14,15], and a member of the senescence and nutrient remobilisation controlling *NAC *gene family [16]. Natural and artificially mutagenised *T. monococcum *populations have been made available and were used to identify and map genes of agronomic importance [17,18]. Furthermore, TILLING (Targeting Induced Local Lesions IN Genomes) and VIGS (Virus Induced Gene Silencing) platforms for functional genomics are under development in several laboratories (; ). Thus, in the foreseeable future *T. monococcum *is expected to play an important role in wheat genetic and genomic studies.

Globally, thousands of accessions of *T. monococcum *have been collected and retained in major germplasm stock centres. In order to use *T. monococcum *resources efficiently in programmes on genetic improvement of hexaploid wheat, it is necessary to assess diversity of this species at the genome level. For this, high-throughput molecular marker technologies are needed. *T. monococcum *has been shown to possess a high level of polymorphism at DNA marker loci[19]. RFLP (Restriction fragment length polymorphism) and AFLP (Amplified fragment length polymorphism) markers have been developed and used for generating genetic linkage maps and for mutation mapping, map-based cloning, and genome synteny comparisons [4,20-23]. These markers have also been used to resolve the site of the *T. monococcum *domestication [1]. Microsatellite markers (also called simple sequence repeats or SSRs) have been tested in *T. monococcum *in comparison with hexaploid wheat and its A-genome donor diploid species *T. urartu*. This information has been used to produce a genetic map integrating RFLPs and SSRs [24,25]. However, the aforementioned markers are primarily gel-based and sequence-dependent. The cost per data point, labour-intensive assay procedures and the limitation of polymorphism of the current marker technologies restricts their application to whole-genome scan approaches such as large-scale genotyping of germplasm collections, association mapping, pedigree analysis and QTL (Quantitative Trait Loci) Mapping As You Go [26].

Diversity Arrays Technology (DArT) has been developed as a sequence-independent and micro-array hybridisation-based marker system [27]. DArT generates medium-density genome scans by scoring the presence versus absence of DNA fragments in representations of genomic DNA samples. It simultaneously determines hundreds to thousands of polymorphic loci in a single assay [27,28]. Since its initial development in rice, DArT has been employed in genetic mapping, genotyping and diversity assessment in barley [28-31], Arabidopsis [32], cassava [33], sorghum [34], hexaploid and durum wheat [35-38], and approximately 30 other plant species (Diversity Arrays Technolgy P/L, unpublished data). DArT has also been used to study pan-genomic evolution in non-model organisms [39] because of its high-throughput and cost-effective nature.

We report here the results of a study aimed to (1) develop a *T. monococcum *diversity array (DArT) for high-throughput genome-wide genotyping, (2) assess the utility of the DArT technology for analysis of genetic diversity in a representative collection of *T. monococcum *accessions, (3) compare the relationships between the *A^m^-*genome and other *Triticum *genomes using DArT markers, (4) produce a genetic linkage map for *T. monococcum *integrating DArT and SSR markers, and (5) refine the genome locations of two morphological trait loci.

## Results

### Array composition

A total of 1536 DArT clones were developed from a *Pst*I*/Taq*I representation generated from a mixture of DNA of two *T. monococcum *accessions MDR002 and MDR308 [35]. These two accessions were the parents for a large mapping population, which had previously been genotyped with SSR markers and used for mapping several agronomically important traits [8,9]. In a parallel project, a substantial number of DArT clones were developed from genomic representations of other *Triticum *species with different ploidy levels (Triticarte P/L, unpublished). We combined clones from all projects to assemble a custom-designed array containing 1536 clones derived from the two *T. monococcum *accessions, 2304 clones derived from hexaploid wheats (including the Triticarte Wheat 2.3 array), 1536 clones derived from tetraploid durum wheat (including the Triticarte Durum 2.0 array), and 1536 clones derived from 15 Iranian accessions of *T. boeoticum *Boiss., which is the wild relative of *T. monococcum *(see Additional file 1; Ali Mehrabi, unpublished data).

### Genetic diversity amongst *T. monococcum *accessions revealed by DArT

The composite array was used to assess the genetic diversity amongst sixteen *T. monococcum *accessions listed in Table 1. They were pre-selected based on their geographical origins, useful traits, available molecular and genetic tools, as well as genetic relationships assessed using SSR fingerprinting [8].

**Table 1:** *T. monococcum *accessions used in this study

**Accession**	**Variety**	**Origin Country**	**Year of collection**	**Growth habit**	**Donors**	**Resources**
MDR001	*flavescens*	Algeria	-	Spring	JIC^6^	Transformable^12^
MDR002	*atriaristatum*	Balkans	-	Spring	JIC	Transformable^12^, mapping population
MDR024	*hornemannii; flavescens*	Chechen	1904	Spring	VIR^7^	
MDR037	*macedonicum*	Armenia	1934	Spring	VIR	
MDR040	*vulgare; macedonicum*	Bulgaria	1940	Spring	VIR	Mapping population
MDR043	*vulgare*	Greece	1950	Spring	VIR	Mapping population
MDR044	*hornemannii*	Turkey	1965	Spring	VIR	Mapping population
MDR045	*vulgare*	Denmark	1970	Spring	VIR	
MDR046	*atriaristatum/macedonicum*	Romania	1970	Spring	VIR	
MDR047	*macedonicum; vulgare*	Hungary	1970	Winter	VIR	
MDR049	*pseudohornemannii*	Iran		Winter	VIR	
MDR050^1^		Italy		Spring	JIC	EMS mutagenised population^13^
MDR217		Turkey		Spring	USDA^8^	Mapping population
MDR229		Spain		Spring	USDA	Mapping population
MDR308^2^		Italy		Spring	UC Davis^9^	BAC library, genetic map, EST library, mapping populations
MDR650^3^		Iran			USDA	
MDR652^4^		Turkey			ACPFG^10^	Mapping populations
MDR657^5^		?			MPI^11^	Mapping populations

^1^Selection from a cross between *T. monococcum *and *T. sinskajae *(Korzun et al., 1998) [42].

^2^*T. monococcum *DV92; provided by Jorge Dubcovsky, UC Davies, USA.

^3^*T. monococcum *PI 355520 from USDA, ARS, USA.

^4^*T. monococcum *AUS16273-2; provided by Dr. Yuri Shavrukov, ACPFG, Australia.

^5^*T. monococcum *L118; provided by Benjamin Killian, MPI, Cologne, Germany.

^6^John Innes Centre, Norwich, United Kingdom.

^7^N. I. Vavilov Institute of Plant Industry, St. Petersburg, Russian Federation.

^8^United States Department of Agriculture, Agricultural Research Service, Aberdeen, ID, USA.

^9^University of California, Davis, CA, USA.

^10^Australian Centre for Plant Functional Genomics, Canberra, Australia.

^11^Max Planck Institute, Cologne, Germany.

^12^Huw Jones, Rothamsted Research, Harpenden, UK, personal communication.

^13^Kay Denyer, John Innes Centre, UK, personal communication.

In total, 846 DArT markers were identified as polymorphic amongst the 16 accessions. Polymorphism Information Content (PIC) values for these markers were relatively low, with only 35.5% of DArTs having PIC values of 0.4-0.5 and 34.6% with PIC values < 0.2 (Table 2). The average mean PIC value was 0.31. When the quality of the DArT markers (measured as the % of total variance in hybridisation intensity between the two clusters: present and absent) was analysed against their performance, which is determined through call rate and PIC values, more than half of the polymorphic DArT markers (*n *= 439) were in the 90-100% quality category with an average PIC value of 0.34 and call rate of 99.8%, respectively (Table 3). The average PIC value decreased with the average quality value. PIC values were > 0.30 when the marker quality was > 80%. However, PIC values were reduced to 0.24, 0.13 and 0.12 for markers with the quality values of 70-80%, 60-70% and 50-60%, respectively.

**Table 2:** Polymorphism information content (PIC) values for 846 DArT markers developed from *T. monococcum *genome.

**PIC value**	**Number of DArTs**	**% total DArTs**
0.5-0.4	300	35.5
0.4-0.3	131	15.5
0.3-0.2	122	14.4
0.2-0.1	161	19
0.1-0.0	132	15.6

**Table 3:** The relationship between the quality and the performance of the 846 DArT markers developed from *T. monococcum *genome.

**Quality (%)**	**100-90**	**90-80**	**80-70**	**70-60**	**60-50**	**Grand mean**
Number of markers	439	237	112	43	15	846
Call rate	99.8 ± 1.2	98.8 ± 2.8	97.9 ± 3.7	99.0 ± 2.5	99.3 ± 2.0	99.2 ± 2.4
PIC	0.34 ± 0.14	0.32 ± 0.14	0.24 ± 0.14	0.13 ± 0.03	0.12 ± 0.02	0.31 ± 0.15

**Figure 1:**
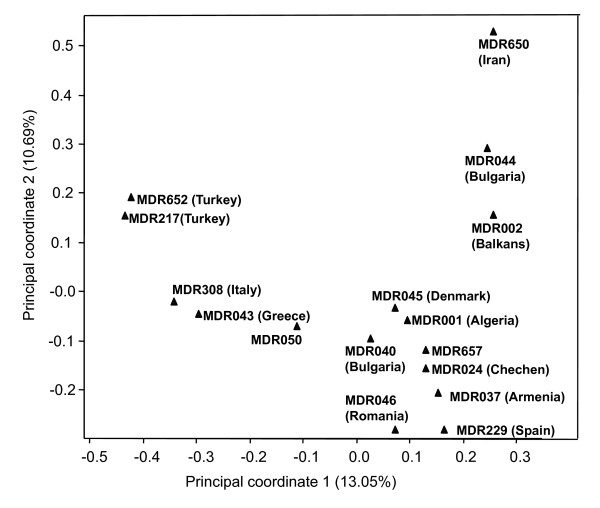
**Principal coordinate analysis of 16 *T. monococcum *accessions based on 846 DArT markers**. The accession codes and their sites of collection are inserted in the figure. The diagram shows the position of each accession in the space spanned by the first two coordinates of a relative Jaccard similarity matrix.

This set of polymorphic DArT markers was used to assess the genetic diversity of the 16 *T. monococcum *accessions. A Jaccard similarity matrix was generated and used to construct a principal coordinate plot deciphering the genetic relationships among the accessions (Figure 1). The first two principal coordinates derived from the scores jointly explained 23.74% of the total data variance. There was a clear separation for most of the *T. monococcum *accessions and partial correlations between genetic relationships and geographic origins. The accession MDR650 (PI 355520) of Iranian origin was fairly distantly related to other accessions including most of the accessions of European origin. This accession is unique in its ability to produce fertile F_1 _hybrids with hexaploid wheat [40]. MDR650 is also a known source of resistance to leaf rust [41] and provides resistance to a range of other wheat pathogens (HCJ and KHK, unpublished). Our previous diversity study using SSR fingerprinting has shown that another Iranian accession MDR049 also formed an independent clade [8]. Furthermore, in the current study MDR308 and MDR043 were clustered close to each other and were distant to MDR002. Amongst the 14 analysed accessions there were two accessions with unusual pedigrees. MDR050 is an inbred line derived from a cross between *T. monococcum *and *Triticum sinskajae *[42], whereas MDR657 (L118) is a recombinant inbred line generated by several rounds of crosses and backcrosses between *Triticum boeoticum *and *Triticum urartu *(B. Kilian, personal communication).

### Relationships between the genomes of *T. monococcum *and related *Triticum spp*

As described above, the custom-designed DArT array contained clones developed from the *T. monococcum *genome and clones derived from the genomes of other *Triticum *species. This allowed us to assess (1) the degree of genetic similarity/diversity between different *T. monococcum *accessions, and (2) the relationships between the genome of *T. monococcum *and the A-genomes of other closely related *Triticum *species. Analysis of the 16 *T. monococcum *accessions revealed that 317 of the 1536 of DArT markers (~20%) developed from *T. monococcum *accessions MDR002 and MDR308 were polymorphic (Table 4). In this set of *T. monococcum *accessions ~10% of DArT markers derived from the genomes of hexaploid and tetraploid wheat were polymorphic, whereas only ~8.2% of DArTs derived from *T. boeoticum *were polymorphic. The latter markers also displayed the lowest PIC values. A similar trend was observed for the polymorphic DArT markers in the F_2 _mapping population derived from MDR308 × MDR002 cross (see below). Overall, the DArT markers developed from *T. monococcum *genome were found to be more informative than those developed from the genomes of other related *Triticum *species.

**Table 4:** Number and feature of polymorphic DArT markers identified in this study

	**Hexaploid wheat**	**Tertraploid wheat**	***T. boeoticum***	***T. monococcum***
Total DArT	2304	1536	1536	1536
				
Polymorphic among 16 accessions	246 (10.68%)	157 (10.22%)	126 (8.20%)	317 (20.64%)
Mean quality (%)	86.3 ± 9.6	86.7 ± 9.3	85.0 ± 10.6	88.7 ± 8.8
Mean call rate (%)	99.3 ± 2.1	99.1 ± 2.1	99.3 ± 2.1	99.1 ± 2.6
Mean PIC	0.30 ± 0.14	0.32 ± 0.15	0.29 ± 0.15	0.32 ± 0.14
				
Polymorphic between MDR002 and MDR308	71 (3.08%)	61 (3.97%)	35 (2.28%)	133 (8.66%)
Mean quality (%)	84.1 ± 6.0	83.3 ± 4.6	82.3 ± 4.9	85.3 ± 5.6
Mean call rate (%)	93.8 ± 3.6	93.5 ± 2.8	93.1 ± 2.7	94.6 ± 3.0
Mean PIC	0.42 ± 0.06	0.43 ± 0.05	0.43 ± 0.04	0.40 ± 0.07

**Figure 2:**
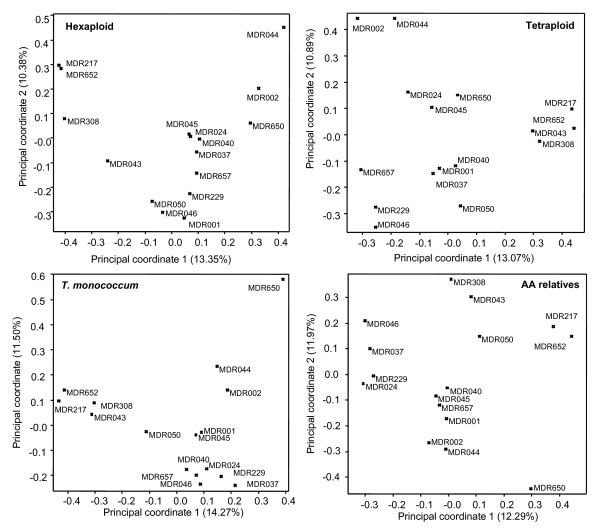
**Principal coordinate analysis of 16 *T. monococcum *accessions based on the DArT markers from *T. monococcum *as well as from diploid, tetraploid and hexaploid *Triticum *species**. The diagrams show the positions of individual accessions in the space spanned by the first two coordinates of a relative Jaccard similarity matrix. The relationships depicted by the four matrices are highly associated as indicated by the Mantel test (see text for details).

To test the power of the DArT markers of different origins in resolving the *T. monococcum *relationships, the 846 polymorphic DArT markers were split according to their genome origins and principal coordinate analyses were carried out using the four subset data. The percentages of total data variance explained by the first two coordinates were 27.75% in hexaploid wheat, 23.96% in tetraploid wheat, 35.26% in genomes of *T. boeoticum*, and 35.77% in *T. monococcum*, respectively (Figure 2). These principal coordinate similarity matrices had a correlation coefficient between 0.73-0.94 in describing the relationships among these *T. monococcum *accessions (Table 5). The Mantel test [43] indicated that the matrices are highly significantly associated (all the *p *= 0.000, less than 0.001).

**Table 5:** Correlation between pairs of similarity matrices describing the relationships of *T. monococcum *accessions generated by using DArT markers from different origins.

	**Whole DArT set**	**Hexaploid**	**Tetraploid**	***T. monococcum***	***T. boeoticum***
Whole DArT set	*				
Hexaploid	0.92 (0.000)	*			
Tetraploid	0.87 (0.000)	0.76 (0.000)	*		
*T. monococcum*	0.94 (0.000)	0.79 (0.000)	0.75 (0.000)	*	
*T. boeoticum*	0.86 (0.000)	0.75 (0.000)	0.73 (0.000)	0.73 (0.000)	*

Numbers between parentheses are the *p*-values for testing the null hypothesis of no association by Mantel test.

**Figure 3:**
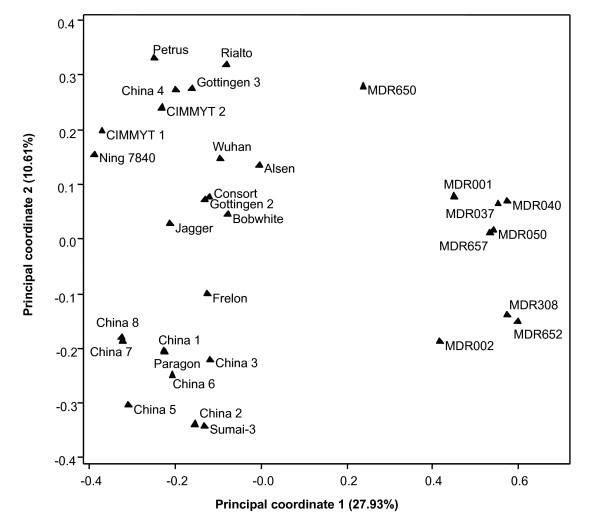
**Principal coordinate analysis of nine *T. monococcum *accessions and 23 *T. aestivum *varieties based on 102 DArT markers**. The diagram shows the position of each accession/variety in the space spanned by the first two coordinates of a relative Jaccard similarity matrix.

To further analyse the relationships of the genomes of *T. monococcum *and those of hexaploid wheat, we simultaneously hybridised the genomes of nine *T. monococcum *accessions and 23 hexaploid wheat varieties of European, American and Chinese origins to a customised Triticarte DArT array (see Additional file 2). The ploidy levels of the genomes did influence the scoring and hence the raw data was analysed to identify DArT markers which were not affected by this genome context-dependent scoring. As shown in Table 6, 1036 DArT markers were reliably scored, of which 696 markers were polymorphic in hexaploid wheat genomes but were commonly present or absent in *T. monococcum *genomes; whilst 238 markers were polymorphic in *T. monococcum *genomes but were monomorphic in hexaploid genomes. However, there were 102 DArT markers which were polymorphic in the genomes of both ploidy. The principal coordinate plot constructed using these DArT markers showed that 38% of the variation was explained by the two-dimensional analysis (Figure 3). The plot showed a good separation of the genomes with different ploidy levels and the genomes within each ploidy level.

**Table 6:** Comparison of DArT markers hybridised to *T. monococcum *and hexaploid wheat genomes

	**Numbers**	**Quality**	**Call Rate**	**PIC**	**Scored in *T. monococcum***	**Scored in hexaploid wheat**	**Score in *T. monococcum***	**Score in hexaploid wheat**
Polymorphic in both ploidy	102	80.5 ± 6.9	96.6 ± 4.1	0.38 ± 0.1	Yes	Yes	0.51 ± 0.32	0.45 ± 0.33
Present in diploid, polymorphic in hexaploid	358	80.3 ± 7.4	97.9 ± 2.7	0.41 ± 0.11	No	Yes	1.00 ± 0.00	0.44 ± 0.27
Absent in diploid, polymorphic in hexaploid	338	82.0 ± 7.0	97.9 ± 2.5	0.44 ± 0.08	No	Yes	0.00 ± 0.00	0.67 ± 0.24
Present in hexaploid, polymorphic in diploid	42	78.3 ± 11.0	96.9 ± 5.7	0.27 ± 0.12	Yes	No	0.41 ± 0.29	1.00 ± 0.00
Absent in hexaploid, polymorphic in diploid	196	82.7 ± 10.0	96.9 ± 5.7	0.26 ± 0.11	Yes	No	0.57 ± 0.28	0.00 ± 0.00

### Genetic mapping of DArT and microsatellite (SSR) loci

To confirm that DArT markers were inherited in a Mendelian manner, we constructed a linkage map for a cross between *T. monococcum *accessions MDR308 and MDR002 [8]. Hybridising the array with a total of 94 F_2 _progeny from this cross as well as the two parental accessions identified 300 polymorphic DArT markers: 133, 71, 61 and 35 were derived from genomes of *T. monococcum*, hexaploid wheat, tetraploid wheat, and *T. boeoticum*, respectively (Table 4). The same *T. monococcum *mapping population was also genotyped using microsatellite markers mapped to the A sub-genome of hexaploid wheat [44]. Out of 274 microsatellite markers analysed, 90 (32.8%) were polymorphic between the two parental *T. monococcum *accessions MDR002 and MDR308. Rates of polymorphism for different types of microsatellite markers were as follows: WMC - 41.9% (36 polymorphic markers out of 86 examined), CFA and CFD - 39.3% (11 out of 28), BARC markers - 38.2% (21 out of 55), GDM and WMS - 33.7% (28 out of 83), and DuPw - 13.6% (3 out of 22). The data for DArT and microsatellite markers were merged for construction of a genetic linkage map for *T. monococcum*.

#### Map length and genome coverage

**Figure 4:**
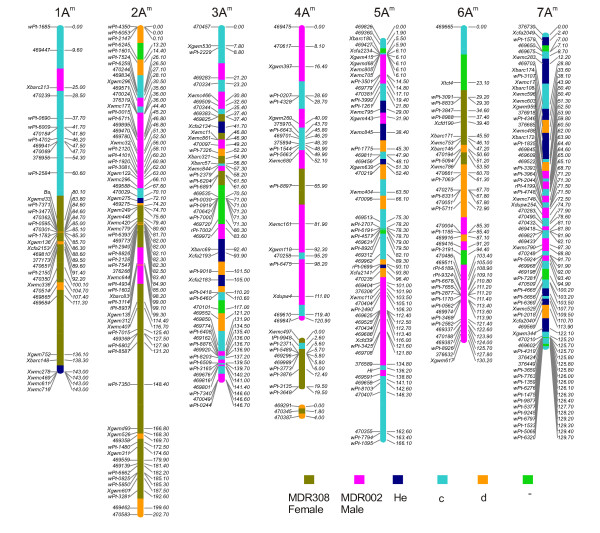
**An integrated DArT and SSR genetic linkage map of *T. monococcum*.** The vertical bars represent the chromosomes of *T. monococcum*. The codes on the left are the DArT and SSR marker loci, with corresponding map locations in accumulative genetic distance (cM; Kosambi) on the right. The discrete segments of the vertical chromosomal bars are colour-coded according to the allele colour in the figure legends. **He **stands for alleles heterozygous for female (MDR308) and male (MDR002) alleles, **c **for the female (MDR308) alleles in homozygous or heterozygous forms, **d **for male (MDR002) alleles in homozygous or heterozygous forms, and **- **for unknown alleles, respectively.

In total, 356 (274 DArTs and 82 SSRs) molecular markers were mapped and formed nine linkage groups (Figure 4). Some DArT and microsatellite markers were removed from the data during the map construction due to a lack of linked anchor markers. The two morphological traits, namely awns colour and leaf hairiness, were found to segregate in a 1:3 ratio in the MDR308 × MDR002 *T. monococcum *mapping population. Therefore, these traits are thought to be controlled by single genes *Ba *(black awn) and *Hl *(hairy leaf) and were also included in the linkage analysis (see below). The linkage map derived from the combined data set spanned 1062.72 cM, with an average length of 151.82 cM per chromosome and an average density of one marker per 2.97 cM. Each of the seven chromosomes contained both DArT and SSR markers. Six of the linkage groups corresponded to six *T. monococcum *chromosomes, but the chromosome 4A^m ^was formed by three linkage groups.

#### Marker distribution amongst chromosomes

Various numbers of DArT and SSR markers were mapped to individual chromosome linkage groups (Table 7). Chromosome 4 A^m ^contained the lowest numbers (*n *= 34) of the molecular markers, while the highest numbers (*n *= 68) were found on chromosome 7 A^m^. Kolmogorov-Smirnov tests were performed to compare nonparametrically the equality of the distributions of the DArT and SSR markers along individual chromosomes. The results showed that chromosomes 1A^m^, 3A^m^, 5A^m^, 6A^m ^and 7A^m ^had *p*-values smaller than 0.05 but chromosomes 2A^m ^and 4A^m ^had larger *p*-values. These results suggest that DArT and SSR tended to form independent clusters on chromosomes in *T. monococcum*.

**Table 7:** Features of a genetic linkage map for *T. monococcum *integrating DArT and SSR markers

	**1A^m^**	**2A^m^**	**3A^m^**	**4A^m^**	**5A^m^**	**6A^m^**	**7A^m^**	**Total**
Total markers	37	69	52	34	56	42	68	358
DArT	25	50	41	27	42	35	54	274
SSR	11	19	11	7	13	7	14	82
Morphological trait locus	1 (*Ba*)				1(*Hl*)			2
								
Kolmogorov-Smirnov test	9.20 (0.011)	3.78 (0.151)	8.11 (0.017)	1.90 (0.386)	10.50 (0.005)	10.08 (0.006)	10.00 (0.007)	
								
Length (cM)	143.02	202.69	146.69	144.4	166.06	130.19	129.67	
								
Density (cM/marker)	3.87	2.94	2.82	4.24	3.01	3.1	1.96	2.97

#### Distribution of DArT markers derived from genomes of different Triticum species

**Figure 5:**
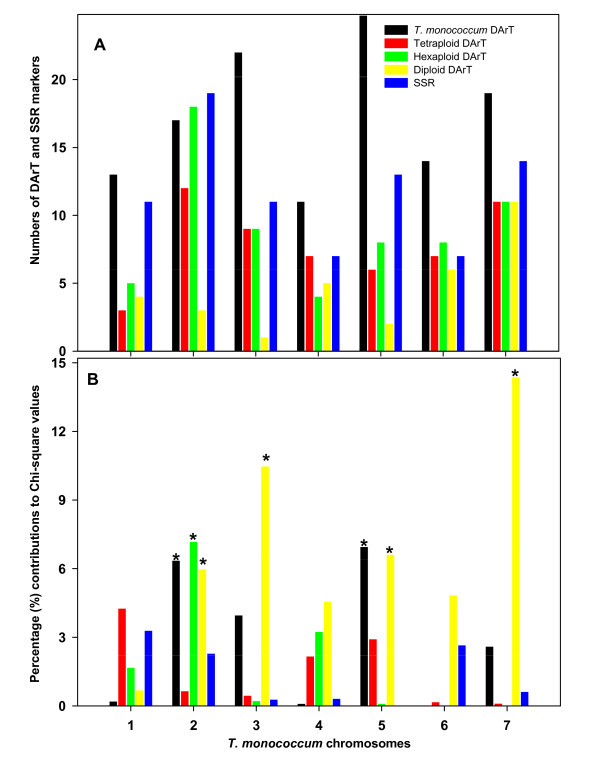
**Chromosome distribution (A) of DArT markers developed from genomes of different *Triticum *species and SSR markers across the seven chromosomes of *T. monococcum *and the percentage (%) contributions (B) of the individual marker categories to the overall Chi-square values (Person χ^2 ^= 27.49 at 34 d. f.; p = 0.778)**. The asterisks indicate over- or under-representations of the numbers of the particular DArT and SSR markers on the chromosomes.

The mapped 274 polymorphic DArT markers were derived from *T. aestivum*, *T. durum*, *T. monococcum *and *T. boeoticum*. Figure 5 shows the numbers of the four category DArT markers and SSR markers on the seven chromosomes. Chi-square goodness-of-fit test of associations between origins of DArT markers and *T. monococcum *linkage groups showed that the distribution of DArT markers arising from different genomes and SSR markers was at random across chromosomes (Pearson χ^2 ^= 27.49 with 34 d.f., probability level under null hypothesis *p *= 0.778). However, in some cases, DArT markers of certain origins were either over- or under-numbered. For instance, the related A genome DArT markers were under-represented on chromosomes 3A^m^, but over-represented on 7A^m^.

Fifty-eight of these markers are with known locations in genomes of hexaploid and tetraploid wheat (Triticarte P/L, unpublished); only 33 are of A-genome origins (Table 8), suggesting that B and D genomes of other *Triticum *species could provide substantial polymorphic information in the A^m ^genome of *T. monococcum*. Each of the seven *T. monococcum *chromosomes carried loci of the DArT markers that were previously mapped to homoeologous chromosomes in the B- or D-genomes of *Triticum *species (Figure 4). Also, the map positions of some of the A-genome derived DArTs determined in our study disagreed with those obtained in previous studies. For instance, some of the markers thought to map to 4A and 7A in hexaploid and tetraploid wheat were mapped to the chromosome 2A^m ^in *T. monococcum*, whereas some other DArTs thought to map to 7A in hexaploid and tetraploid wheat were mapped to either 4A^m ^or 5A^m ^in our study (Table 8, and Figure 4).

**Table 8:** The distribution of DArT markers originated from genomes of various *Triticum *species across the seven chromosomes of *T. monococcum*.

**Marker Name**	**A^m ^map position**	**A^m ^Chromosome**	**Original chromosome**	**Source**	**Clone ID**
wPt-1685	0	1A^m^	1D	A-Genome	375942
wPt-7371	84.63	1A^m^	1B	Durum wheat	379045
wPt-3477	84.65	1A^m^	1B	Bread wheat	119840
wPt-0595	85.02	1A^m^	1A|1B	Durum wheat	346277
wPt-1782	85.26	1A^m^	1A|1B	A-Genome	376064
wPt-2150	91.4	1A^m^	1A|2B	Bread wheat	119519
wPt-6053	0.05	2A^m^	2B	Bread wheat	120879
wPt-1601	14.37	2A^m^	7A	Durum wheat	381522
wPt-7524	26.15	2A^m^	4A	Bread wheat	116494
wPt-1920	60.94	2A^m^	2B	Durum wheat	408383
wPt-3114	98.95	2A^m^	2A	Bread wheat	115722
tPt-8937	99.09	2A^m^	2A	Durum wheat	348413
wPt-7015	125.41	2A^m^	3B	Bread wheat	116612
wPt-6802	127.86	2A^m^	3B	Bread wheat	119652
wPt-7350	148.43	2A^m^	2B	Bread wheat	116096
wPt-1480	172.52	2A^m^	2A	Bread wheat	116703
wPt-6662	182.22	2A^m^	2A	Bread wheat	120517
wPt-3281	192.61	2A^m^	2A	Bread wheat	115316
wPt-7326	52.25	3A^m^	1A	Durum wheat	408336
wPt-2379	58.15	3A^m^	4D	Bread wheat	116321
wPt-6204	61.54	3A^m^	3A	Bread wheat	120579
wPt-6891	61.56	3A^m^	3A	Bread wheat	120585
wPt-6460	110.58	3A^m^	7A	Bread wheat	120067
wPt-8876	136.71	3A^m^	3A	Bread wheat	121186
wPt-6509	139.48	3A^m^	3D	Durum wheat	345122
wPt-3165	139.7	3A^m^	3D	Bread wheat	116398
wPt-7340	146.65	3A^m^	3A|3B	Durum wheat	377884
wPt-0244	146.69	3A^m^	3A|3B	Durum wheat	305793
wPt-6643	45.84	4A^m^	2B	Durum wheat	373941
wPt-8897	65.87	4A^m^	7A	Bread wheat	116046
wPt-2371	127.59	4A^m^	7A	A-Genome	376548
wPt-1261	21.8	5A^m^	5B|5D	Bread wheat	120208
wPt-2707	78.22	5A^m^	5B	Bread wheat	120752
wPt-4577	78.71	5A^m^	5B	Bread wheat	116733
wPt-8920	79.54	5A^m^	7B	Bread wheat	116434
wPt-3425	121.62	5A^m^	7A	Durum wheat	380762
wPt-3091	29.23	6A^m^	6A	Bread wheat	116120
wPt-8833	29.26	6A^m^	6A|6B	Bread wheat	115618
wPt-7063	61.3	6A^m^	6A	Bread wheat	115260
wPt-0562	113.39	6A^m^	6A	Durum wheat	345110
wPt-3468	113.93	6A^m^	6A	Bread wheat	116359
wPt-2582	121.36	6A^m^	6A	A-Genome	376551
wPt-3107	38.13	7A^m^	3B	Bread wheat	116406
wPt-3393	68.69	7A^m^	7A	Bread wheat	119701
wPt-3964	71.2	7A^m^	7A	Durum wheat	305423
wPt-2044	71.29	7A^m^	7A	Durum wheat	305067
wPt-4748	71.48	7A^m^	7A	Bread wheat	115379
rPt-4199	71.48	7A^m^	7A	Durum wheat	347395
wPt-7281	93.37	7A^m^	1A|7A	Durum wheat	343649
wPt-4319	125.75	7A^m^	7B|7D	A-Genome	376425
wPt-7763	125.99	7A^m^	7A|7D	Bread wheat	116340
wPt-1359	126.04	7A^m^	7B|7D	A-Genome	376448
wPt-9877	126.42	7A^m^	7B	Durum wheat	346285
wPt-1533	129.34	7A^m^	7B	Bread wheat	117080
wPt-5069	129.4	7A^m^	7B	Bread wheat	116930
wPt-6320	129.67	7A^m^	7B	Bread wheat	116539

#### Segregation distortion

**Figure 6:**
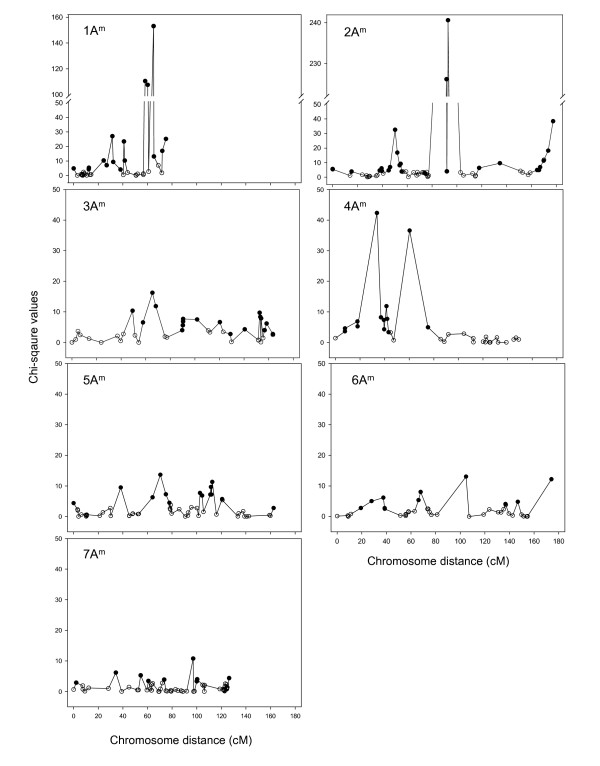
**The distribution of χ^2 ^values for individual DArT and SSR loci as a function of the genetic linkage map along the chromosomes in *T. monococcum***. The genetic loci with open symbols fit 1:2:1 or 1:3 ratios of allele segregation frequencies, whereas those with close symbols showed segregation distortion.

In total, 156 markers were significantly distorted from the expected Mendelian segregation ratios (*P *< 0.05). These were more or less equally distributed across the genome (Figure 6). The chromosomes 1A^m^, 2A^m^, 3A^m^, 4A^m^, 5A^m^, 6A^m ^and 7A^m ^contained 37.8% (17/37), 43.5% (30/69), 46.2% (24/52), 38.2% (13/34), 30.4% (17/56), 30.9% (13/42) and 14.7% (10/68) of markers displaying strong allelic frequency distortion, respectively. For some markers the segregation distortion was in favour of alleles originating from the male parent MDR002, whereas for other markers the segregation distortion was in favour of alleles originating from the female parent MDR308 (Figure 4). The predominant alleles on the chromosomes 1A^m ^and 2A^m ^were from MDR308, whereas those on 4A^m ^were from MDR308. Chromosomes 3A^m^, 4A^m^, 5A^m^, 6A^m ^and 7A^m ^contained marker alleles originating from either male of female parents, displaying frequencies skewed from their Mendelian expectations.

### Fine-mapping of morphological trait loci

In *T. monococcum*, black awns and hairy leaves are two morphological traits known to be controlled by single dominant genes *Ba *and *Hl*, respectively. Our previous genetic study revealed a linkage between *Ba *and the SSR locus *Xwmc336 *on 1A^m^, and between *Hl *and the SSR locus *Xcfd39 *on 5A^m ^[8]. These two traits segregated in MDR002 × MDR308 F_2 _mapping population as single independent loci and therefore it was possible to refine the positions of *Ba *and *Hl *in *T. monococcum *genome. Previous estimations showed that the genetic distance was 21.5 cM between *Ba *and *Xwmc336*, and 17.4 cM between *Hl *and *Xcfd39*. Linkage analysis combining SSR and DArT markers confirmed that *Ba *was located at 80.09 cM on 1A^m ^between the DArT marker locus *wPt-2584 *at 60.59 cM and the SSR locus *Xgwmd33 *at 83.76 cM, and *Hl *was located at 136.16 cM on 5A^m ^between the DArT marker loci *376589 *at 134.84 cM and *469591 *at 138.77 cM.

## Discussion

The availability of a high density genetic linkage map for a species is essential for identifying QTLs of interest, isolation of genes by map-based cloning, comparative mapping, and genome organisation and evolution studies [45]. We have developed polymorphic DArT markers from genome representations of two *T. monococcum *accessions, 15 *T. boeoticum*, and hexaploid and tetraploid *Triticum *species. The genetic linkage map of *T. monococcum *constructed in this study is the first map integrating DArT and SSR markers.

### Determining genetic relationships between *T. monococcum *accessions and with hexaploid wheat varieties using DArT marker fingerprinting

DArT markers are typically developed from a representation which is generated from a pool of DNA samples from a number of accessions, cultivars or breeding lines which as a group cover the genetic diversity within a species or a group of closely related species [27]. In the current study, DArT markers were initially generated from genomic representations of two *T. monococcum *accessions, parents of an F_2 _mapping population. In addition, this study took advantage of the existence of a large number of DArT markers previously developed from hexaploid, tetraploid and other diploid *Triticum *species.

The inclusion of DArT clones from various genomes meant the average PIC value of the data set (0.31) was lower than in comparable studies. For example, for barley, sorghum and cassava the values obtained were 0.38, 0.41 and 0.42, respectively [28,33,34]. However, the data provided useful information for comparison of *Triticum *genomes of different ploidy levels. In the genetic diversity assessment of 16 *T. monococcum *accessions, 20.6% (317 out of 1536) of the DArT markers developed from the two *T. monococcum *accessions were polymorphic. DArT markers developed from genomes of other *Triticum *species also displayed good polymorphism frequencies (10.68%, 10.22% and 8.2% for markers developed from hexaploid, tetraploid and diploid species, respectively) in *T. monococcum*. In hexaploid wheat, 15.3% (788 out of 5137) of DArT markers were found to be polymorphic when assessing the genetic diversity of 13 Australian cultivars [35]. Similar studies in durum wheat and barley revealed only 9.6% and 10.4% of polymorphic DArTs, respectively [28,36]. Thus, the group of 16 *T. monococcum *accession genotyped in this study appeared to be more genetically diverse than the bread and durum wheat collections assayed in those studies. Furthermore, most of the DArT markers whose genome locations have been determined in previous studies were located on homoeologous chromosomes of *T. monococcum*. Thus, DArT markers from related genomes were also useful in probing genetic diversity in *T. monococcum*. The custom-designed DArT array developed here can be used in studies focusing on comparison of the *T. monococcum *genome with genomes of other *Triticum *species.

Principal coordinate analysis of the 16 *T. monococcum *accession revealed that the site of collection is only partially correlated with genetic diversity. Most accessions used in this study have been genotyped with SSR markers in a previous study, and a similar pattern had been observed [8]. Thus, DArT fingerprints are as useful for resolving the genetic relationships in *T. monococcum *as non-DArT based markers. One of the accessions, MDR650 (PI 355520) was found to be genetically distantly related to other *T. monococcum *accessions, which raised a question whether it is a true *T. monococcum*. However, MDR650 (PI 355520) possesses the three major traits of the domesticated einkorn wheat: larger and plumper seeds, a tough rachis preventing spikelets falling apart at maturity, and relatively easy threshing. These traits are absent in the wild species *T. boeoticum*. We have crossed MDR650 (PI 355520) with many other *T. monococcum *accessions and discovered that the crossability could reach 100% (data not shown). Furthermore, MDR650 (PI 355520) did not cluster with hexaploid wheat and was closer to the *T. monococcum *cluster. Thus, we still consider MDR650 (PI 355520) an accession of *T. monococcum*.

The relationships between the A^m^-genome of *T. monococcum *and those of hexaploid wheat *T. aestivum *and its sub-genome donor, *T. urartu *have been studied in different context. For instance, chromosome pairing and recombination were studied between homoeologous chromosomes 1A and 1A^m^, 3A and 3A^m ^as well as 5A and 5A^m ^and were shown to be collinear and differentiated at sub-structural level [20,46]. Good micro-colinearity/colinearity at particular genetic loci has also been reported [47,48]. Other studies as well as the present one demonstrate that many A-genome molecular markers could also be used for genetic studies in *T. monococcum *[24,25]. However, their genome-wide use was not previously reported. The use of DArT markers allowed us to assess the relationships in a new perspective. We showed here that most of the DArT markers which are polymorphic in *T. monococcum *were either conserved or absent in hexaploid genomes, and *vice versa*, indicative of divergence of the A^m^-genome from the A-genome [25]. On the other hand, many DArT markers originated from B- and D-genomes of hexaploid wheat could hybridise to the *T. monococcum *genomes and provide the polymorphism information in *T. monococcum*. Thus, at the homoeologous genomes level, there is a complex relationship between *T. monococcum *and hexaploid wheat.

### *T. monococcum *genetic linkage maps

A *T. monococcum *genetic map has been previously constructed using MDR308 (DV92) as one of parental accessions by Dubcovsky et al (1996) [21]. This map contains 335 markers, including RFLPs, isozymes, seed storage proteins, rRNA, and various morphological loci. The total length of this map is 1071.6 cM, the average chromosome length is 153.1 cM, and the average marker density is one marker per 3.2 cM. More recently, Singh et al (2007) [24] used a *T. boeoticum *× *T. monococcum *RIL population to construct another genetic linkage map integrating 177 SSR and RFLP markers, and two morphological trait loci. The total length of this map is 1262 cM, the average chromosome length is 180.3 cM, and the average marker density is one marker per 7.05 cM. In comparison, our current genetic map derived from linkage analysis of an F_2 _population from a cross between MDR308 and MDR002 integrates 274 DArT markers, 82 SSR markers and two morphological trait loci. This map spans over 1062.7 cM, with six chromosomes represented by single linkage groups and chromosome 4A^m ^by three groups of linked markers. The average chromosome length is 151.8 cM, and the average marker density in the genome is one marker per 2.97 cM. Observed differences in lengths and marker densities for the three *T. monococcum *linkage maps may well be related to differences in the mapping populations, the types and error-rates of the genetic marker systems used, and/or the algorithms and mapping functions used to compute genetic distances. For example, Singh et al (2007) [24] used the Haldane's mapping function, whereas the Kosambi's mapping function was used in other cases.

The current genetic linkage is also very similar in length and in marker densities to the A-genome maps of hexaploid wheat constructed using the Kosambi's map function. In one case, 369 SSR markers were mapped onto a 944 cM A-genome with the marker density of one marker per 2.56 cM [44]. In another study, 464 SSR markers were mapped onto a 1231 cM A-genome with the marker density of one marker per 2.65 cM [49]. Recently DArT markers have also been developed and integrated with conventional markers i.e. SSRs, RFLPs and AFLPs in hexaploid wheat [35] and tetraploid wheat [36].

### Genome organisation and segregation distortion

The A^m^-genome of *T. monococcum* is closely related to the A^u^-genome of *T. urartu* and to the A-genome of the hexaploid wheat [4-6]. This is reflected by the high transferability of SSR markers from hexaploid wheat to *T. monococcum *and the overall good colinearity of SSR arrangement along the chromosomes [8,24]. Gaps are frequently observed in hexaploid wheat genetic linkage maps making use of SSR, RFLP and AFLP markers [49-52]. Similarly, Singh et al (2007) [24] observed four large gaps on linkage maps of chromosomes 2A^m^, 4A^m ^and 7A^m ^in *T. monococcum*. In contrast, in our current linkage map for *T. monococcum *there was only two gaps on chromosome 4A^m^. This suggests that DArT markers can be used to fill the gaps and help generate higher resolution genetic linkage maps.

Strong segregation distortion was observed during construction of our current genetic linkage map for *T. monococcum*. Over one third of the marker loci across the seven chromosomes displayed allele frequencies skewed from their Mendelian expectations. This was observed for both DArT and SSR markers, with preferences for both parental alleles. Segregation distortion has also been observed by Dubcovsky et al (1996) [21] and Singh et al (2007) [24] during construction of einkorn wheat genetic linkage maps, regardless of the type of populations used. In one study (Dubcovsky et al 1996) [21] 15% of the marker loci displayed segregation distortion and were clustered on chromosomes 1A^m ^and 7A^m^, while in other study (Singh et al 2007) [24] two major distorted regions were detected on chromosome 2A^m^. Strong segregation distortion has also been noted during the construction of durum wheat and bread wheat linkage maps integrating SSR and DArT markers [36].

### Utility of DArT markers and the genetic linkage map in *T. monococcum*

The two parental *T. monococcum *lines, MDR308 and MDR002, used for developing the F_2 _mapping population had a number of contrasting traits including awn colour, leaf pubescence, grain hardness, salt tolerance, as well as resistance to the fungus *Mycosphaerella graminicola *and to soil-borne cereal mosaic viruses [8,9,53]. We demonstrated here that the constructed genetic linkage map helped refine the chromosome regions spanning the *Ba *and *Hl *trait loci. Also, this map was recently used to refine the *TmStb1 *locus conferring resistance to *M. graminicola *isolate IPO323 on chromosome 7A^m^, and to identify and map new QTLs conferring salt tolerance (HCJ and KHK, unpublished).

MDR308 (also known as DV92), one of the parental lines of the F_2 _mapping population used in this study, has previously been used for developing a range of molecular tools including a genetic linkage map integrating RFLPs, AFLPs and seed storage proteins [54], a BAC library [55], and several populations of chemically- and radiation-induced mutants . The availability of DArT marker information for MDR308 (DV92) further increases the utility of this accession.

In the near future it should be possible to link various available genetic linkage maps of *T. monococcum *thereby creating a frame work consensus map for wider applications in molecular genetics and genomics studies.

## Conclusion

DArT is a rapid and efficient approach to develop many new molecular markers for genetic studies in *T. monococcum*. The constructed genetic linkage map will facilitate localisation and map-based cloning of genes of interest, comparative mapping as well as genome organisation and evolution studies between this ancient diploid species and other crops.

## Methods

### Plant material

This study used 16 *T. monococcum *accessions and an F_2 _population of 98 individuals derived from a cross between accessions MDR002 and MDR308 [8]. The detailed information about these accessions is also listed in Table 1. In addition, 23 hexaploid wheat varieties collected from UK, continental Europe, China and America, or developed at CIMMYT, Mexico were used for genetic diversity compassion. The information about these hexaploid wheat varieties is provided in Additional file 2.

### DArT procedure

The DNA was extracted from leaves of 2-week-old einkorn wheat seedlings using the DNeasy Plant Mini Kit (QIAGEN) according to manufacturer's instructions. A set of 1536 new DArT clones were generated from a *Pst*I/*Taq*I representation of the MDR002 and MDR308 accessions as described previously [28,36]. The new clones were printed together with 2304 polymorphism-enriched clones from hexaploid wheat, 1536 from tetraploid wheat, and 1536 from a group of 15 Iranian accessions of diploid *T. boeoticum *(see Additional file 1). The resulting composite array was then used to genotype the *T. monococcum *samples using the standard DArT protocol [28,36].

### Microsatellite assay

A total of 279 SSR markers mapped to the A-genome of hexaploid wheat were tested for polymorphism between the *T. monococcum *accessions MDR002 and MDR308. These SSRs originated from 5 groups: 57 BARC markers from the Beltsville Agricultural Research Centre, USA [56,57], 29 CFA and CFD markers from INRA Clermont-Ferrand, France [52,58,59], 24 DuPw markers from DuPont company ([60] et al. 2002, DuPont, unpublished; Dreisigacker et al. 2005), 85 GDM and WMS markers from IPK Gatersleben, Germany [61,62], and 89 WMC markers from the Wheat Microsatellite Consortium [63]. For each SSR primer pair, the 5'-end of the forward primer was labelled with infra-red dye (IRD700 or IRD800, LI-COR Biosciences UK Ltd). The PCR were carried out using the PCR Master Mix (Promega) in a 10-μl reaction volume containing 1× PCR Master Mix buffer, 0.1 μM forward and reverse primers, and 20-30 ng plant DNA. Amplifications were carried out in 96-well microtiter plates using a G-storm Thermal Cycler (GS4/GS4s, Gene Technologies, Essex, England). The programmes were 2 min at 94°C, followed by 30-35 cycles of 30 s at 94°C, 30 s annealing at 50-60°C (depending on the primer pairs), and 1 min at 72°C, and a final extension of 5 min at 72°C. The final PCR products were diluted to 20-40 times using formamide Li-Cor loading dye, denatured for 5 min at 85°C and stored on ice before 0.5-0.8 μ1 of the reaction mix was loaded on Li-Cor 4300 DNA Analyser.

### *T. monococcum *accession diversity analysis and comparison with hexaploid wheat

A group of 16 *T. monococcum *accessions were genotyped using the custom-built DArT array as described previously [36]. The polymorphic DArT markers were scored, and used for clustering analysis using principal coordinate plots. For genome comparison, genome representations of nine *T. monococcum *accessions and 23 hexaploid wheat varieties were hybridised to a newly built DArT array and scored the same way. The ploidy levels did influence the scoring as envisaged in the raw data of the hybridisation intensity. Therefore, only the DArT markers which were not affected by this context-dependent scoring were scored. The nine *T. monococcum *accessions and 23 hexaploid wheat varieties were hybridised in duplicate.

### Genetic mapping and integration of DArT and SSR markers

*T. monococcum *accessions MDR308, MDR002 and 94 F_2 _individuals derived from a cross between them were genotyped with the custom-built array as described previously [36]. In total, 300 DArT markers, 90 SSR markers as well as two morphological trait loci (*Ba*, black awn; *Hl*, hairy leaf) were used for genetic linkage map construction using JoinMap^® ^4.0 (Van Ooijen, J.W., 2006, JoinMap^® ^4.0, Software for the calculation of genetic linkage maps in experimental populations. Kyazma B. V., Wageningen, The Netherlands). Because of the inherent dominant nature of DArT markers which separates the female and male markers in the repulsion phase, the linkage maps for maternal DArT markers (scored as *a*, *c*) and for parental DArT markers (scored as *b*, *d*) were initially constructed independently with SSR markers. The two maps for individual chromosomal linkage groups were then merged together using SSR markers as bridges. Assignment of markers to linkage groups was achieved using logarithm of the odds (LOD) threshold values ranging from 3.0 to 10.0. The Kosambi's map function was used to estimate genetic distances. In total, 358 markers including 274 DArT markers, 82 SSR markers and 2 morphological trait locus markers were integrated into nine linkage groups. Goodness of fit for all the loci to an expected 1:2:1 or 1:3 segregation ratio was tested using chi-square (*χ*^2^) analysis. The graphical representation of the map was drawn using GGT2.2 software [64].

### Statistical analysis

All statistical analyses were carried out using GenStat (10^th ^edition, VSN International, UK). For principal coordinate analysis Jaccard similarity matrices were generated using the DArT markers. Two-dimensional scores were calculated and used to generate scatter plot matrices of scores. For the Mantel test which looks for association between the off-diagonal values of two similarity (or distance) matrices, the correlation of the matrices were evaluated. The DArT markers were subdivided into four different categories depending on their genome origins and similarity matrices calculated. The similarity coefficients from these matrices were then compared pair by pair to form a new correlation matrix. The significance of each correlation was assessed using a randomisation test (with 1000 random permutations). The *p*-values were calculated and were always < 0.001 (where the null hypothesis is one of zero association). For the Kolmogorov-Smirnov test, the map position data of the DArT and SSR markers on individual chromosomes were fed into GenStat and the *p*-values were calculated. The Chi-square goodness-of-fit test was carried out to examine the random distribution of DArT markers of different origins across the genome, by calculating the association between the numbers of the markers of different origins the chromosomes.

### Data deposition

The genetic marker and linkage map data, along with details and accession numbers for the deposition of raw data, are freely available at the UK WGIN  and Monogram  as well as the GrainGene  websites.

## Competing interests

Employees of DArT PL co-authoring this paper (AK, EH and PW) provide DArT array commercial genotyping services for a range of crops and may benefit financially from this work.

## Authors' contributions

All authors read and approved the final manuscript. KHK, HCJ and AK designed the study and coordinated the research activities. HCJ, KHK and KK drafted the manuscript. HCJ, CB, KK and SB developed SSR markers for *Triticum monococcum*. PW, EH and AK developed the DArT array for *T. monococcum*, mapped DArT markers, and edited the manuscript. KHK raised the funds, initiated and supervised the whole project.

## Supplementary Material

Additional file 1 The 15 Iranian accessions of *Triticum boeoticum *used to generate the customised DArT array used in this study. Click here for file

Additional file 2 Hexaploid wheat varieties used in comparative analysis using DArT markers. Click here for file
