# Supplementary material for: DArT markers: diversity analyses, genomes comparison, mapping and integration with SSR markers in Triticum monococcum
Source: BMC Genomics. 2009 Sep 30;10:458. doi: 10.1186/1471-2164-10-458 (PMC2764732; doi:10.1186/1471-2164-10-458)
Supplement: Additional file 1 — The 15 Iranian accessions of Triticum boeoticum used to generate the customised DArT array used in this study. [file 1471-2164-10-458-S1.doc]

The 15 Iranian accessions of *Triticum boeoticum* used to generate the customised DArT array used in this study

| Organism | Genotype | Tissue |
| --- | --- | --- |
| Wheat  Wheat  Wheat  Wheat  Wheat  Wheat  Wheat  Wheat  Wheat  Wheat  Wheat  Wheat  Wheat  Wheat  Wheat | 132  133  134  135  136  137  138  140  142  143  144  145  146  147  164 | leaf  leaf  leaf  leaf  leaf  leaf  leaf  leaf  leaf  leaf  leaf  leaf  leaf  leaf  leaf |
