# Supplementary material for: DArT markers: diversity analyses, genomes comparison, mapping and integration with SSR markers in Triticum monococcum
Source: BMC Genomics. 2009 Sep 30;10:458. doi: 10.1186/1471-2164-10-458 (PMC2764732; doi:10.1186/1471-2164-10-458)
Supplement: Additional file 2 — Hexaploid wheat varieties used in comparative analysis using DArT markers. [file 1471-2164-10-458-S2.doc]

Hexaploid wheat varieties used in comparative analysis using DArT markers

|  | **Variety** | **Also known as** | **Origin** |
| --- | --- | --- | --- |
| V1 | Piko |  | Germany |
| V2 | Petrus |  | Germany |
| V3 | Centrum |  | Germany |
| V4 | Greif |  | Germany |
| V5 | Astron |  | Germany |
| V6 | Consort |  | UK |
| V7 | Rialto |  | UK |
| V8 | Maris Huntsman |  | UK |
| V9 | Soissons |  | France |
| V10 | Frelon |  | France |
| V11 | Sumai-3 |  | China |
| V12 | Ning 7840 |  | China |
| V13 | Wuhan |  | China |
| V14 | USA1 | Jagger | USA |
| V15 | Bobwhite |  | Mexico (CIMMYT) |
| V16 | Gottingen 2 |  | Germany |
| V17 | Gottingen 3 |  | Germany |
| V18 | CIMMYT 1 | CIMMYT 29* | Mexico (CIMMYT) |
| V19 | CIMMYT 2 | CIMMYT 30* | Mexico (CIMMYT) |
| V22 | Paragon |  | UK |
| V23 | Bobwhite |  | Mexico (CIMMYT) |
| V24 | China 3 | Z-9023 | China |
| V25 | China 4 | Wanmai-27 | China |
| V26 | China 7 | N962390 | China |
| V27 | China 6 | Wan9926 | China |
| V28 | China 1 | Wanmai-43 | China |
| V29 | Alsen |  | USA |
| V30 | China 2 | Sumai-3 | China |
| V31 | China 5 | Ningmai-8 | China |
| V32 | China 8 | Yang 158 | China |

* the provenance of both Cimmyt lines has been lost
